# Associations of free sugars from solid and liquid sources with cardiovascular disease: a retrospective cohort analysis

**DOI:** 10.1186/s12889-023-15600-3

**Published:** 2023-04-24

**Authors:** Kaberi Dasgupta, Joseph Mussa, Anne-Sophie Brazeau, Mourad Dahhou, Claudia Sanmartin, Nancy A. Ross, Elham Rahme

**Affiliations:** 1 Department of Medicine, McGill University, Montreal, QC Canada; 2 Centre for Outcomes Research and Evaluation (CORE), Research Institute of the McGill University Health Centre (RI-MUHC), 5252 Boul de Maisonneuve Ouest, Office 3E.09, Montreal, QC H4A 3S5 Canada; 3 School of Human Nutrition, McGill University, Montreal, QC Canada; 4 Statistics Canada, Division of Health Analysis, Ottawa, ON Canada; 5 Department of Public Health Sciences, Queen’s University, Kingston, ON Canada

**Keywords:** Heart disease, Stroke, Added sugars, Intake limits

## Abstract

**Background:**

The World Health Organization recommends a 10% total energy (TE%) limit for free sugars (i.e., added sugars and naturally occurring sugars in fruit juice, honey, and syrups) based on evidence linking higher intakes with overweight and dental caries. Evidence for cardiovascular disease (CVD) is limited. Impacts may differ by sex, age group, and solid vs. liquid sources; liquids may stimulate more adverse CVD profiles (due to their rapid absorption in the body along along with triggering less satiety). We examined associations of consuming total free sugars ≥ 10 TE% with CVD within four sex and age-defined groups. Given roughly equal free sugar intakes from solid and liquid sources, we also evaluated source-specific associations of free sugars ≥ 5 TE% thresholds.

**Methods:**

In this retrospective cohort study, we estimated free sugars from 24-h dietary recall (Canadian Community Health Survey, 2004–2005) in relationship to nonfatal and fatal CVD (Discharge Abstract and Canadian Mortality Databases, 2004–2017; International Disease Classification-10 codes for ischemic heart disease and stroke) through multivariable Cox proportional hazards models adjusted for overweight/obesity, health behaviours, dietary factors, and food insecurity. We conducted analyses in separate models for men 55 to 75 years, women 55 to 75 years, men 35 to 55 years, and women 35 to 55 years. We dichotomized total free sugars at 10 TE% and source-specific free sugars at 5 TE%.

**Results:**

Men 55 to 75 years of age had 34% higher CVD hazards with intakes of free sugars from solid sources ≥ 5 TE% vs. below (adjusted HR 1.34, 95% CI 1.05- 1.70). The other three age and sex-specific groups did not demonstrate conclusive associations with CVD.

**Conclusions:**

Our findings suggest that from a CVD prevention standpoint in men 55 to 75 years of age, there may be benefits from consuming less than 5 TE% as free sugars from solid sources.

**Supplementary Information:**

The online version contains supplementary material available at 10.1186/s12889-023-15600-3.

## Background

The appropriate ceiling for sugar intakes is debated, particularly in relationship to cardiovascular disease (CVD). The World Health Organization (WHO) advocates an upper limit of 10% of total energy consumed (TE%) for free sugars, which includes both added sugars and sugars naturally present in fruit juice, honey, and syrups [1, 2]. This is based on systematic reviews demonstrating associations of sugar consumption with obesity and dental caries [2]. However, while obesity is an established risk factor for CVD, [3] evidence for direct associations between sugar intakes and CVD remains limited.

In the 1950s, some epidemiological and mechanistic studies [4] suggested associations between sugar intakes and CVD, but this area of work was suppressed for many years by the sugar industry and collaborating academic researchers [4]. More studies have been conducted since that time, but the United Kingdom's Scientific Advisory Committee on Nutrition [5] and the German Nutrition Society's [6] reviews of the evidence determined it to be inconclusive. In contrast, the Heart and Stroke Foundation of Canada recommended that less than 10 TE% be derived from added sugars, based on an American study [7] linking National Health and Nutrition Examination Survey (NHANES) and mortality data; Americans who consumed 10 to 25 TE% as added sugar were 30% more likely to experience a fatal CVD event than those consuming less than 10 TE%.

Added sugars average approximately 17 TE% in the United States, [7] more than the 10 TE% average intake reported in Canadian surveys [8]. Our objective was to examine associations of consumption of free sugars at or above 10 TE% with CVD using Canadian data. We were also interested in assessing for differences in associations for solid vs. liquid sugar sources. While the energy provided by a given quantity of free sugars is source-independent, solid foods are digested more slowly than liquids, with a generally lower glycemic index, and thus possibly less likely to stimulate adverse cardiometabolic profiles [9]. There is also evidence of earlier satiety with sugar-sweetened solids than sugar-sweetened liquids, thereby limiting intake [10–12]. Finally, the ‘added sugars’ portion of free sugars consists primarily of fructose in beverages and sucrose in foods; [13] fructose may have particularly deleterious impacts on hepatic metabolism and thereby insulin resistance, [9, 14] with potential effects on CVD development. Given that consumption from solid and liquid sources of free sugars is roughly equally divided, [15] we separately examined free sugars from solid and from liquid sources, each above vs. below a 5 TE% threshold. We evaluated a combined endpoint of both CVD-related mortality and hospital admissions for CVD-related events.

In addition, men and women differ importantly in health behaviours and underlying CVD risk, [16, 17] as do younger to middle-aged vs. older adults; thus, impacts of free sugars may differ in relationship to sex and age. Indeed, as proposed by Woodward, [18] sex disaggregation should be the norm in CVD research, given differences in biological and social factors. Such factors may also broadly differ between younger and older adults. We therefore conducted sex and age-category specific analyses.

## Methods

Our retrospective cohort study included estimates of free sugars derived from 24-h dietary recall of the 2004–2005 Canadian Community Health Survey (CCHS). Our outcome was nonfatal and fatal CVD, with diagnoses extracted from the national hospital Discharge Abstract Database (DAD) and Canadian Mortality Database (CMDB) for the 2004–2017 period. This study was conducted according to the Strengthening the Reporting of Observational studies in Epidemiology (STROBE) guideline.

### Ethics

The Health Canada Research Ethics Board approved the CCHS. Each participant completed written informed consent procedures, with a separate consenting procedure for linkage to DAD and CMDB. Statistics Canada linked 95% of CCHS responses to DAD data in consenting individuals [19, 20]. The Social Sciences and Humanities Research Council of Canada reviewed and approved the analyses presented herein (17-SSH-MCG-5265) which we conducted on deidentified data at the secure access sites of the Quebec Inter-University Center for Social Statistics operated by Statistics Canada.

### Data sources

CCHS (multistage stratified cluster design) is a biennial national evaluation to gain an understanding of the health status of Canadians. It includes both a recurring interviewer-administered questionnaire and cycle-specific components, which in 2004–2005 included direct height and weight measures and a 24-h dietary recall (United States Department of Agriculture Automated Multiple-Pass Method) [21]. Statistics Canada linked these with DAD and CMDB, using probabilistic methods based on common identifiers (names, date of birth, postal code, sex) [20]. DAD records information on hospitalizations (mandatory submission), in all provinces and territories except Quebec; it includes International Classification of Disease (ICD), 10^th^ revision-coded diagnoses and admission and discharge dates of hospitalization. The CMDB includes ICD-coded causes of death and dates of death, for all events since 1950 (mandatory reporting).

### Study population

Among all CCHS 2004–2005 respondents who agreed to data linkage, we focused on those 35 to 75 years old at baseline, arguably old enough to experience CVD events in sufficiently large numbers and young enough that dietary factors might have some impact not overridden by age effects [22]. The index date was the specific date of completion of the 2004–2005 CCHS cycle 2.2 interview. We excluded those with prior CVD (CCHS responses indicating prior CVD and/or ICD-10 codes for myocardial infarction, angina, or stroke on DAD between 1999 and 2003). We also excluded those who reported diabetes of any type on the CCHS, given possible impacts on consumption of free sugars. Follow-up was from index date to first CVD event (fatal or nonfatal ischemic heart disease or stroke), death from other causes, or December 14, 2017 (the end of the observation period).

### Free sugars

Statistics Canada grouped 24-h recall dietary items and amounts into Bureau of Nutritional Sciences (BNS) food and beverage categories. Health Canada originally developed BNS categories in order to summarize more detailed food and recipe information included in nutrition surveys [23]. Statistics Canada cross-referenced BNS categories with the Canadian Nutrient File to estimate TE, total sugars, and other dietary factors. We separated consumed items into solid and liquid groups. We ascertained the grams of free sugars consumed from each item by multiplying the Statistics Canada-computed total sugars amount by Bernstein and colleagues’ estimates [24] of % free sugars for each BNS category. We converted this to kcals (multiplying by 4 kcal/gram), and then divided by TE intake during the recall period, such that free sugars were expressed as TE%. We computed free sugars from solids, from liquids, and from both combined (total free sugars).

Thirty percent of CCHS respondents completed a second 24-h dietary recall, which was performed 3 to 10 days after the first interview, on a different day of the week [21]. For this subgroup, we ascertained the proportions classified as being ≥ 5 TE% for free sugars from solid sources and from liquid sources (separately) and as being ≥ 10 TE% for free sugars from solid and liquid sources (combined). We examined Cramer’s V between first and second recalls, as well as proportions switching categories (i.e., moving from above threshold to below and vice versa).

### Other baseline characteristics

We defined obesity as body mass index (BMI) ≥ 30 kg/m^2^ and overweight as BMI 25–30 kg/m^2^ [25]. When direct measures were unavailable, we used self-reported values and applied a previously established correction factor (BMI_measured_ =  − 0.12 + 1.05*BMI_self-reported_) [26]. We abstracted the following dietary covariates: fats (saturated, monounsaturated and polyunsaturated), protein, sodium, potassium, fibre, non-sugar carbohydrates, and TE intake from the 24-h dietary recall component of the CCHS survey. We also considered daily intake of 5 or more servings of fruit and vegetables and daily fruit juice intake (*How often do you usually drink fruit juices such as orange, grapefruit or tomato?)* from the general questionnaire. We examined demographic (age, ethnicity [Europid vs. non-Europid], immigrant status, food insecurity, urban–rural residence, educational level) and health behaviours (physical activity [active vs. inactive if < 1.5 metabolic equivalents/kg/ day], current smoking) derived from the general questionnaire, as well as self-assessment of food intake compared to usual intake (much more, typical, much less) from the 24-h recall. The CCHS general questionnaire also queried whether participants had been diagnosed by a health professional with ‘high blood pressure,’ ‘cancer,’ ‘a bowel disorder such as Crohn’s disease or colitis,’ or ‘intestinal/stomach ulcers’; we considered these co-morbid conditions. We did not have information on dyslipidemia, prediabetes, or specific biomarkers.

### Outcomes

For CVD ascertainment, we applied ICD-10 codes to DAD and CMDB for ischemic heart disease (I20-25) and stroke (I60, I61, I63, I64, H340-H341, G45) as well as Canadian Classification of Health Intervention codes for procedures related to percutaneous coronary intervention, coronary artery bypass graft surgery, cardiac catheterization, and thrombectomy including carotid artery procedures [27]. ICD-10 codes for myocardial infarction [28] and stroke [29] in hospitalization have previously been validated.

### Analyses

We used SAS version 9.3 (SAS Institute, Inc., Cary, North Carolina). We computed baseline characteristics (TE; free sugars from liquids, from solids, and total; overweight and obesity status; dietary co-variates; health behaviours; intake relative to usual; co-morbid conditions), dividing the study population into four demographic groups: men 55 to 75 years, women 55 to 75 years, men 35 to 55 years, and women 35 to 55 years old. We do not report on cell counts with fewer than 30 people, as per Statistics Canada’s policy for protection of confidentiality. We ascertained the top solid and liquid BNS categories from which free sugars were consumed.

We constructed Cox proportional hazards models to examine associations of free sugars from solids and free sugars from liquids at or above vs. below the 5 TE% threshold, with fatal and nonfatal CVD, separately for each of the four demographic groups defined. We constructed separate models for each of these sex and age groups because of potential effect modification by age group and/or sex. For each of the four age-group and sex-specific models, we adjusted for age as a continuous variable as the age range within each model spanned 20 years. When evaluating free sugars from solid sources and free sugars from liquid sources, both were included in a given model (each expressed categorically as at or above 5 TE% vs. below) and total sugars were not included.

We adjusted models for baseline characteristics (age as a continuous variable, ethnicity [Europid vs. non-Europid], immigrant status, food insecurity, urban–rural residence, educational level, overweight/obesity, physical activity, smoking status, self-assessment of food intake compared to usual intake, high blood pressure, Crohn’s disease/colitis, ulcers, cancer, daily intake of 5 or more servings of fruit and vegetables, daily fruit juice intake, and dietary covariates (fats [saturated, monounsaturated and polyunsaturated], protein, sodium, potassium, fibre, non-sugar carbohydrates, and TE intake in kcals). In the main models, we opted to include a full macronutrient profile. However, given scope for overadjustment, we also present models that exclude macronutrients other than free sugars and non-sugar carbohydrates. We evaluated impacts on hazard ratios (HRs).

We used a similar approach to examine associations of total free sugars (at or above 10 TE% vs. below) with fatal and nonfatal CVD; these models were adjusted for baseline characteristics but did not include free sugars from solid sources and/or free sugars from liquid sources as separate variables. We assessed for multicollinearity among covariates (Cramer’s V for pairwise comparisons between categorical covariates; Pearson’s correlation coefficient for pairwise comparisons between continuous covariates; point-biserial correlation coefficient for pairwise comparisons between categorical and continuous covariates) and tested the proportional hazards assumption through Schoenfeld’s residuals (for individual covariates and the global test).

While we made an a priori decision to evaluate free sugars as at or above vs. below defined thresholds, we did assess for linear relationships between free sugars and the time-to-event outcome, through visual examination of scatter plots to which we applied locally estimated scatterplot smoothing (LOESS; PROC SGSSCATTER). LOESS generates a curve that best fits the distribution of data.

With respect to the four demographic groups that we separately analyzed, when point estimates were similar in magnitude and direction for both sexes of a given age group, we decided a priori that we would collapse categories. Because the direction of association was similar for men and women 55 to 75 years old, we created a combined model for this age group, additionally adjusted for sex. We did not combine men and women 35 to 55 years of age as HR point estimates differed importantly.

## Results

**Fig. 1:**
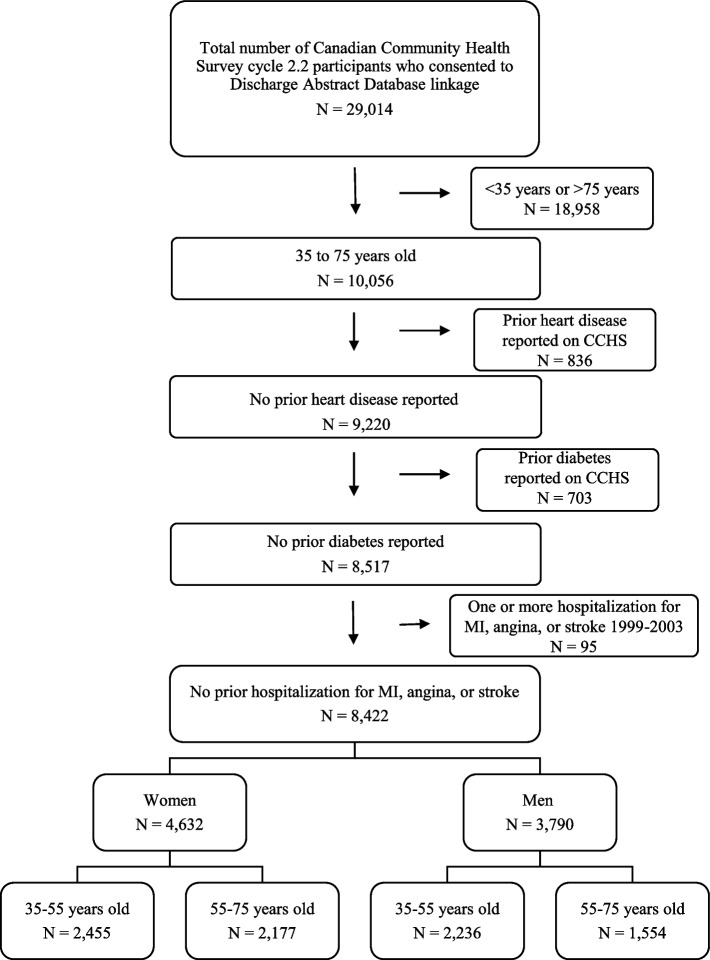
Cohort construction. Legend: The study cohort was constructed by applying eligibility criteria to the Canadian Community Health Survey Cycle 2.2 participants who consented to data linkage with the Discharge Abstract Database and the Canadian Mortality Database. CCHS Canadian Community Health Survey; MI Myocardial infarction

Among CCHS respondents who permitted data linkage (29,104; Fig. 1), approximately one third were 35 to 75 years of age (10,056), among whom 84% remained (8,422) after exclusions for diabetes and/or CVD. The study population consisted of 18% men 55 to 75 years of age (1554) and 25 to 30% in each of the other demographic groups (women 55 to 75 years- 2,177; men 35 to 55 years- 2,236; women 35 to 55 years- 2,455) who were followed up over a median of approximately 13.5 years (interquartile range, 13.3–13.6 years). We did not detect multicollinearity (Cramer’s V < 0.10; Pearson’s correlation coefficient <|0.30|; point-biserial correlation coefficient <|0.30|, for all pairwise comparisons, indicating weak correlation) and Schoenfeld’s test was not statistically significant (*p* > 0.05), for each of the covariates or globally.

### Free sugars at baseline

Average values for free sugars ranged between 10.3 and 11.5 TE% (SD 7.6 to 9.3; Table 1). Free sugars from solid sources averaged 5 to 5.6 TE% while corresponding values from liquid sources were 4.6 to 6.6 TE%.

**Table 1:** Baseline characteristics, separated by sex and age group

	**55 to 75 years**		**35 to 55 years**	
**Baseline characteristics**^a^	**Men** ***N* = 1554**	**Women** ***N* = 2177**	**Men** ***N* = 2236**	**Women** ***N* = 2455**
Overweight, number (%)^b^	735 (48.9)	790 (37.9)	939 (43.3)	748 (32.1)
Obesity, number (%)^b^	385 (25.6)	609 (29.2)	626 (28.9)	655 (28.1)
BMI^a^, kg/m^2^, mean (SD)^b^	27.8 (4.6)	28.0 (6.0)	28.1 (5.1)	27.7 (6.6)
Age, years, mean (SD)	63.8 (6.3)	64.2 (6.3)	45.0 (5.6)	45.2 (5.7)
Europid, number (%)	1462 (94.1)	2049 (94.2)	2000 (89.5)	2177 (88.7)
Immigrant, number (%)	281 (18.1)	349 (16.1)	251 (11.3)	280 (11.4)
Food insecure, number (%)	48 (3.1)	92 (4.2)	167 (7.5)	252 (10.3)
Rural residence, number (%)	463 (29.8)	576 (26.5)	613 (27.4)	617 (25.1)
Post-secondary education, number (%)	822 (52.9)	1053 (48.4)	1418 (63.4)	1580 (64.4)
Current smoker, number (%)	351 (22.6)	386 (17.7)	785 (35.1)	756 (30.8)
Active, number (%)	736 (47.4)	969 (44.5)	964 (43.1)	1031 (42.0)
Total energy intake kcal, mean (SD)	2194.3 (913.4)	1638.1 (665.0)	2446.2 (1073.3)	1749.4 (763.2)
Amount of food intake reported in last 24-h compared to usual intake, number (%)	Much more: 84 (5.4)	Much more: 166 (7.6)	Much more: 138 (6.2)	Much more: 210 (8.6)
	Typical: 1277 (82.3)	Typical: 1713 (78.8)	Typical: 1704 (76.3)	Typical: 1765 (72.0)
	Much less: 191 (12.3)	Much less: 296 (13.6)	Much less: 391 (17.5)	Much less: 477 (19.5)
**Free Sugar Intakes: % of total energy, mean (SD)**				
Overall free sugars	10.4 (7.6)	10.3 (7.9)	11.5 (9.3)	11.1 (9.0)
Free sugars in solids	5.6 (4.7)	5.7 (4.8)	5.0 (4.7)	5.7 (5.5)
Free sugars in liquids	4.7 (6.3)	4.6 (6.3)	6.6 (8.1)	5.4 (7.4)
Non-sugar carbohydrates	27.4 (8.5)	28.0 (9.2)	27.1 (9.1)	27.4 (9.8)
**Fats and Proteins: % of total energy, mean (SD)**				
Saturated fats	10.4 (4.4)	10.0 (4.3)	10.4 (4.1)	10.2 (4.4)
Monounsaturated fats	12.9 (4.6)	12.3 (4.7)	13.0 (4.6)	12.7 (4.8)
Polyunsaturated fats	5.7 (2.7)	5.8 (2.8)	5.6 (2.6)	5.7 (2.8)
Protein	16.8 (5.9)	16.8 (5.8)	16.9 (6.6)	16.6 (6.2)
**Other, mean (SD)**				
Sodium, g	4.2 (2.6)	3.2 (1.9)	4.5 (2.9)	3.4 (2.1)
Potassium, g	4.4 (2.4)	3.6 (2.0)	4.4 (2.5)	3.6 (2.0)
Fibre, g	24.2 (16.4)	20.9 (13.8)	22.7 (15.3)	19.5 (13.2)
Daily fruit juice, number (%)	660 (42.5)	986 (45.3)	909 (40.7)	897 (36.5)
≥ 5 servings of fruit and vegetables daily, number (%)	423 (27.3)	812 (37.5)	473 (21.2)	756 (30.9)
**Co-morbid conditions**				
Hypertension	403 (25.9)	706 (32.4)	218 (9.75)	234 (9.53)
Intestinal/stomach ulcers	39 (2.51)	78 (3.58)	53 (2.37)	82 (3.34)
Cancer	66 (4.25)	62 (2.85)	17 (0.76)	34 (1.38)
Bowel Disorders	32 (2.06)	101 (4.64)	36 (1.61)	118 (4.81)

The mean intake of free sugars ranged between 10.3 and 11.5 TE% (SD 7.6 to 9.3; Table 1). Free sugars from solid sources averaged 5 to 5.7 TE% while corresponding values from liquid sources were 4.6 to 6.6 TE%. Across each demographic group, more than 70% of participants reported that the 24-h recall reflected their typical daily consumption

*BMI* Body mass index, *SD* Standard deviation

^a^Men and women with missing data for covariates other than overweight/obesity (i.e. Europid, immigrant, post–secondary education, etc.) were collectively unavailable for < 1% of the study cohort (data not shown)

^b^BMI computation was based on directly measured anthropometric data in 61% of men 55 to 75 years, 67.5% of women 55 to 75 years, 59% of men 35 to 55 years, and 63% of women 35 to 55 years. BMI data was missing for 3.2% of men 55 to 75 years, 4.3% of women 55 to 75 years, 3% of men 35 to 55 years, and 5.2% of women 35 to 55 years; these individuals were excluded from the regression models. For the remainder, BMI was computed using self–reported data. Overall, there was under 5% missingness across all variables

With respect to top sources of free sugars among solids (Table 2), nearly half of participants reported consumption of seasonings, sugar, or spice added to food (means across groups: 25% consumed items from seasonings category, 16 to 18% from white or brown sugar category, 6% from spices category); 15% consumed items from the bread product category (5 to 6% white bread, 3.5 to 5% whole wheat bread, 3.4 to 5% from other bread products). For liquids, roughly 45% of participants consumed items from sauces, fruit juice, and salad dressing categories (means: 11.5 to 18% consumed sauces [e.g., white, béarnaise, soya, tartar, ketchup], 13 to 19% fruit juice, 13 to 17% salad dressing); 13% of free of men 35 to 55 years of age consumed soft drinks, higher than the mean 5 to 8% reported from the other demographic groups.

**Table 2:** Percent of participants consuming one or more items from various Bureau of Nutritional Sciences (BNS) categories of items that include free sugars

**Top Solid Sources Of Free Sugars** (BNS item, %FS, % reporting)	**Top Liquid Sources Of Free Sugars** (BNS item, %FS, % reporting)
Men 55–75 Years	
1. Seasonings (salt, pepper, etc.) – 86%FS – 25%	1. Fruit juice – 100%FS – 19%
2. Sugars (white and brown) – 100%FS – 17.5%	2. Salad dressings – 91%—13%
3. White bread – 56%FS – 6.2%	3. Sauces (white, bearnaise, soya, tartar, ketchup, etc.) – 65%FS – 13%
4. Spices – 86%FS – 6.1%	4. Other sugars (syrups, molasses, honey, etc.) – 100%FS – 12.5%
5. Whole wheat breads – 56%FS – 4.9%	5. Soft drinks – regular – 100%FS- 7.8%
6. Rolls, bagels, pita bread, croutons, dumplings, matzo, tortilla – 56%FS – 4.8%	6. Soups without vegetables – 52%FS – 5.7%
7. Canned tomatoes – 15%FS – 3.7%	7. Half&half creams – 73%FS – 5.5%
8. Whole grain, oats and high fibre breakfast cereals – 76%FS – 2.2%	8. Vinegar – 12%FS – 4.9%
9. Jams, jellies and marmalades – 100%FS – 2.1%	9. Fruit drinks – 100%FS – 3.4%
10. Cookies, commercial – 100%FS – 2.1%	10. Other beverages (malted milk, instant breakfast) – 70%FS -2.9%
Women 55–75 Years	
1. Seasonings (salt, pepper, vinegar, etc.) – 86%FS – 25%	1. Fruit juice – 100%FS – 18%
2. Sugars (white and brown) – 100%FS – 15.5%	2. Salad dressings – 91%—17%
3. Spices – 86%FS – 6%	3. Sauces (white, bearnaise, soya, tartar, ketchup, etc.) – 65%FS – 11.5%
4. Whole wheat breads – 56%FS – 6%	4. Other sugars (syrups, molasses, honey, etc.) – 100%FS – 9.8%
5. White bread – 56%FS – 5%	5. Soups without vegetables – 52%FS – 8%
6. Whole grain, oats and high fibre breakfast cereals – 76%FS – 4.6%	6. Soft drinks – regular – 100%FS- 5%
7. Rolls, bagels, pita bread, croutons, dumplings, matzo, tortilla – 56%FS – 3.4%	7. Vinegar – 12%FS – 4.9%
8. Canned tomatoes – 15%FS – 3.3%	8. Fruit drinks – 100%FS – 4.6%
9. Cookies, commercial – 100%FS – 2.7%	9. Half&half creams – 73%FS – 4.6%
10. Jams, jellies and marmalades – 100%FS – 2.3%	10. Other beverages (malted milk, instant breakfast) – 70%FS -3.7%
Men 35–55 Years	
1. Seasonings (salt, pepper, vinegar, etc.) – 86%FS – 25%	1. Sauces (white, bearnaise, soya, tartar, ketchup, etc.) – 65%FS – 18.7%
2. Sugars (white and brown) – 100%FS – 17.5%	2. Fruit juice – 100%FS – 13%
3. White bread – 56%FS – 6%	3. Soft drinks – regular – 100%FS- 13%
4. Spices – 86%FS – 6%	4. Salad dressings – 91%—12.5%
5. Canned tomatoes – 15%FS – 4.9%	5. Other sugars (syrups, molasses, honey, etc.) – 100%FS – 7.8%
6. Rolls, bagels, pita bread, croutons, dumplings, matzo, tortilla – 56%FS – 4.8%	6. Soups without vegetables – 52%FS – 5.7%
7. Whole wheat breads – 56%FS – 3.7%	7. Half&half creams – 73%FS – 5.5%
8. Cookies, commercial – 100%FS – 2.2%	8. Fruit drinks – 100%FS – 4.9%
9. Whole grain, oats and high fibre breakfast cereals – 76%FS – 2.1%	9. Other beverages (malted milk, instant breakfast) – 70%FS -3.4%
10. Cheese, 10% b.f. to 25% b.f. – 1.9%	10. Vinegar – 12%FS – 2.9%
Women 35–55 Years	
1. Seasonings (salt, pepper, vinegar, etc.) – 86%FS – 25%	1. Sauces (white, bearnaise, soya, tartar, ketchup, etc.) – 65%FS – 16%
2. Sugars (white and brown) – 100%FS – 16%	2. Fruit juice – 100%FS – 15%
3. Spices – 86%FS – 6%	3. Salad dressings – 91%—14%
4. White bread – 56%FS – 4.6%	4. Other sugars (syrups, molasses, honey, etc.) – 100%FS – 8%
5. Canned tomatoes – 15%FS – 4.5%	5. Soft drinks – regular – 100%FS- 7.5%
6. Rolls, bagels, pita bread, croutons, dumplings, matzo, tortilla – 56%FS – 4.5%	6. Soups without vegetables – 52%FS – 6%
7. Whole wheat breads – 56%FS – 3.9%	7. Other beverages (malted milk, instant breakfast) – 70%FS -5.5%
8. Whole grain, oats and high fibre breakfast cereals – 76%FS – 2.7%	8. Half&half creams – 73%FS – 5%
9. Chocolate bars – 100%FS – 2.3%	9. Vinegar – 12%FS – 4.8%
10. Cookies, commercial – 100%FS – 2.2%	10. Fruit drinks – 100%FS – 4%

The top solid sources of free sugar were seasonings, sugar, or spice (added to food), and bread products. The top liquid sources of free sugar were sauces, fruit juice, and salad dressings. Younger men (35–55 years of age) more frequently reported the consumption of soft drinks relative to the other demographic groups

*BNS* Bureau of Nutritional Sciences, b.f. butter fat, *%FS* Percent of total sugars component calculated to be free sugars

In the study cohort, 2,352 (27.9%) completed a second 24-h dietary recall. There was a moderate correlation between recalls for proportion classified as ≥ 5 TE% for free sugars from both solid sources (Cramer’s V = 0.22) and liquid sources (Cramer’s V = 0.32), with 39% switching categories for solid sources and 33% for liquid sources. There was a similar correlation for those classified as ≥ 10 TE% for free sugars from solid and liquid sources combined (Cramer’s V = 0.25), with 38% switching.

### Other baseline characteristics

Over 88% of participants were Europid (European origin) with nearly 90% of younger people and over 80% of older people born in Canada. More than 60% of the younger people and roughly half of the older people reported postsecondary education.

Approximately a quarter of participants were in the obesity category (Table 1), with less than 50% categorized as physically active. Food insecurity was highest in younger women (10.3%) and lowest in older men (3.1%). Approximately one third of the younger people and one fifth of older people smoked cigarettes. Under 10% of younger people, more than a quarter of older men, and over a third of older women reported hypertension.

More than 70% across groups reported that the 24-h recall reflected their usual intake (Table 1). Total energy averaged 1,600 to 1,700 kcal in women and approximately 2,000 to 2,500 kcal in men. Non-sugar carbohydrate comprised roughly one quarter of energy intakes, similar to total fats. Approximately 17% of TE intake were from proteins across all demographic groups. Sodium intake averaged 3.2 to 3.4 g in women and 4.2 to 4.5 g in men. Fibre intake was about 20 g in women and somewhat higher in men. Consumption of 5 or more servings of fruit and vegetables daily occurred in more women (younger women, 30.9%; older women, 37.5%) than men (younger men, 21.2%; older men, 27.3%). Over one third to nearly one-half of participants consumed fruit juice daily.

### CVD event rates

CVD event rates were highest in older men (Table 3), with event rates in older women at just over half of the average rate in men. Event rates were much lower in younger men and younger women. In all demographic groups except younger men, event rates were higher in those consuming free sugars at or above the 5 TE% threshold for solid sources and for liquid sources and in those consuming total free sugars at or above the 10 TE% threshold examined (Table 3).

**Table 3:** CVD incidence rates and adjusted hazard ratios in relationship to free sugars above vs. below defined thresholds^a^

	CVD events, N	Person-years of follow-up	CVD incidence rate (events/1000 person-years)	Unadjusted HR (95% CI)	Adjusted HR (95% CI)^b^ including only free sugars and non-sugar carbohydrates among dietary co-variates	Adjusted HR (95% CI)^c^ with broader group of dietary co-variates
**Solid Sources Of Free Sugars And Cvd Across 5 TE% Threshold**						
***Men, 55 to 75 years***						
< 5 TE%	166	9,309	17.8	1.13 (0.91 – 1.41)	**1.23** **(1.02 – 1.55)**	**1.34** **(1.05** – **1.70)**
≥ 5 TE%	158	7,884	20.0			
***Women, 55 to 75 years***						
< 5 TE%	141	13,534	10.4	1.16 (0.92 – 1.46)	1.08 (0.84 – 1.37)	1.10 (0.85 – 1.43)
≥ 5 TE%	151	12,571	12.0			
***Men and women, 55 to 75 years***^d^						
< 5 TE%	307	22,843	13.4	1.13 (0.97 – 1.32)	**1.14** **(1.01** – **1.35)**	**1.21** **(1.02** – **1.44)**
≥ 5 TE%	309	20,455	15.1			
***Men, 35 to 55 years***						
< 5 TE%	95	17,318	5.49	0.95 (0.69 – 1.32)	0.88 (0.62 – 1.23)	0.90 (0.63 – 1.28)
≥ 5 TE%	59	11,426	5.16			
***Women, 35 to 55 years***						
< 5 TE%	32	17,696	1.81	1.52 (0.95 – 2.41)	1.55 (0.93 – 2.5)	1.54 (0.90 – 2.62)
≥ 5 TE%	40	14,589	2.74			
**Liquid Sources Of Free Sugars And CVD Across 5 TE% Threshold**						
***Men, 55 to 75 years***						
< 5 TE%	198	11,094	17.9	1.15 (0.92 – 1.44)	1.06 (0.83 – 1.35)	1.13 (0.87 – 1.47)
≥ 5 TE%	126	6,102	20.7			
***Women, 55 to 75 years***						
< 5 TE%	188	17,436	10.8	1.12 (0.88 – 1.42)	1.10 (0.84 – 1.42)	1.09 (0.82 – 1.45)
≥ 5 TE%	104	8,696	12.0			
***Men and women, 55 to 75 years***^d^						
< 5 TE%	386	28,530	13.5	1.15 (0.98 – 1.36)	1.07 (0.90 – 1.28)	1.12 (0.92 – 1.35)
≥ 5 TE%	230	14,798	15.5			
***Men, 35 to 55 years***						
< 5 TE%	88	15,581	5.65	0.88 (0.64 – 1.22)	0.99 (0.70 – 1.40)	0.90 (0.62 – 1.31)
≥ 5 TE%	65	13,183	4.93			
***Women, 35 to 55 years***						
< 5 TE%	39	20,482	1.90	1.47 (0.92 – 2.33)	1.55 (0.92 – 2.62)	1.26 (0.71 – 2.24)
≥ 5 TE%	33	11,815	2.79			
**Total Free Sugars And CVD Across 10 TE% Threshold**						
***Men, 55 to 75 years***						
< 10 TE%	169	9380	18.0	1.10 (0.88 – 1.37)	1.08 (0.86 – 1.36)	1.18 (0.91 – 1.53)
≥ 10 TE%	155	7816	19.8			
***Women, 55 to 75 years***						
< 10 TE%	160	15,137	10.6	1.15 (0.91 – 1.44)	1.19 (0.93 – 1.53)	1.24 (0.93 – 1.67)
≥ 10 TE%	132	11,009	12.0			
***Men and women, 55 to 75 years***^d^						
< 10 TE%	329	24,517	13.4	1.14 (0.97 – 1.34)	1.11 (0.94 – 1.32)	**1.20** **(0.99** – **1.45)**
≥ 10 TE%	287	18,825	15.2			
***Men, 35 to 55 years***						
< 10 TE%	85	14,755	5.76	0.87 (0.63 – 1.19)	0.96 (0.68 – 1.34)	0.89 (0.62 – 1.31)
≥ 10 TE%	69	14,017	4.92			
***Women, 35 to 55 years***						
< 10 TE%	30	17,755	1.69	1.71 (1.07 – 2.73)	**1.76** **(1.05 – 2.97)**	1.43 (0.79 – 2.58)
≥ 10 TE%	42	14,556	2.89			

*TE%* Percent total energy intake on 24-h recall, *N* Number, CVD cardiovascular disease

^a^Free sugars from solid sources and from liquid sources are separately examined above and below a 5 TE% while total free sugars are examined across a 10 TE%, the recommended upper limit for added sugars in several recommendations. The 5 TE% threshold was selected for solid and liquid sources as these sources are roughly equally represented in intakes. Combined men and women models are additionally adjusted for sex. Overall, there was under 5% missingness across all variables entered the regression models; those with missing data were excluded from our regression models

^b^The model was adjusted for normal/overweight/obesity status, age, ethnicity, immigrant status, food insecurity, urban/rural residence, physical activity, educational level, smoking status, self-assessment of food intake compared to usual, non-sugar carbohydates, daily fruit juice intake, having 5 or more servings of fruit and vegetables, and co-morbid conditions

^c^This model is adjusted for all the co-variates indicated for the first adjusted model and also includes saturated fats, monounsaturated fats, polyunsaturated fats, protein, sodium, potassium, fibre or total energy intake

^d^Men and women 55 years and older were also considered together in a combined analysis given that both groups had point estimates suggesting increased risk across the thresholds examined

### Associations between free sugars from solid sources and CVD

For free sugars from solid sources, men 55 to 75 years of age who consumed at or above 5 TE% had 34% greater hazards for fatal or nonfatal CVD than men consuming below this threshold (adjusted HR 1.34, 95% CI 1.05- 1.70; Table 3). Findings were not conclusive in other groups considered in separate models (women 55 to 75 years: HR 1.10, 95% CI 0.85–1.43; women 35 to 55 years HR 1.54, 95% CI 0.90–2.62; men 35 to 55 years HR 0.90, 95% CI 0.63–1.28).

### Associations between free sugars from liquid sources and CVD

For free sugars from liquid sources, no conclusive associations were observed for consumption above vs. below a 5 TE% threshold (men 55 to 75 years, HR 1.13, 95% CI 0.87–1.47; women 55 to 75 years, HR 1.09, 95% CI 0.82–1.45; men 35 to 55 years, HR 0.90, 95% CI 0.62–1.31; women 35 to 55 years HR 1.26, 95% CI 0.71–2.24).

### Associations between total free sugars and CVD

No conclusive associations were observed for consumption above vs. below a 10 TE% threshold for total sugars across the four age ranges and sex-specific models.

### Assessment for linear relationships

Scatterplots of total free sugars, free sugars from solid sources, and free sugars from liquid sources vs. the time-to-event outcome demonstrated departures from linearity (not shown).

### Evaluation for overadjustment related to dietary variables

When we reran models excluding all of the macronutrients other than free sugars and non-sugar carbohydrates, the HRs with respect to free sugars did not change importantly.

### Associations when men and women 55 to 75 years of age were combined

Directions of HRs were similar for men and women 55 to 75 years of age. When men and women 55 to 75 years were included in a single model additionally adjusted for sex, we estimated that those who consumed at or above 5 TE% as free sugars from solid sources had 21% greater hazards for CVD (HR 1.21, 95% CI 1.02–1.44). For total free sugars, men and women who consumed at or above 10 TE% had 20% greater hazards for CVD (HR 1.20, 95% CI 0.99–1.45) although a null effect could not be excluded given that the lower bound of the confidence interval was 0.99.

## Discussion

In our analyses of a Canadian cohort, all associations evaluated in younger age groups (35 to 55 years of age) were inconclusive, likely linked to a relatively low number of overall CVD events. We identified a 34% increased CVD hazards in middle aged to older men (55 to 75 years of age) when 5 TE% or more was in the form of free sugars from solid sources, compared to consumption below this level. In women of this age group, the effect estimate was similar but not conclusive; however, when men and women were combined, the CVD hazards were conclusively 21% higher. Moreover, in this combined group, there was also a signal for a 20% increase in CVD hazards with consumption of total free sugars at or above 10 TE% vs. below this threshold.

This signal for a 20% higher hazards for fatal or nonfatal CVD is consistent with a 30% increased hazards for fatal CVD reported in a previously cited American NHANES analysis [7] for consumption of 10 to 25 TE% as total added sugars compared to below 10 TE%. In contrast, there were no conclusive differences for consumption of added sugars at or above 10 TE% compared to below this level in terms of CVD hazards in a recent Swedish study among middle to older aged men and women [30]. This suggests differences in ability to ‘tolerate’ free sugars in different populations.

Similarly, in our analyses, consumption of free sugars at or above 5 TE% from solid sources in men and women 55 to 75 years of age was associated with 21% greater hazards for CVD compared to consumption below this level, but in the Swedish study, there was actually higher CVD hazards with consumption of less than two ‘treats’(solids) per week compared to greater consumption of treats [30]. One possibility for differences between North America and Sweden is that protective factors in Sweden, such as perhaps higher physical activity or better dietary quality, may be able to partially offset any adverse CVD effects of added sugars up to a certain level. In Sweden, that level may be 20 TE% for total added sugars; in the Swedish study, those consuming at or above 20 TE% had 31% increased hazards of stroke and 39% higher hazards for coronary artery disease than those consuming 5 to 10 TE%. There were insufficient numbers of participants with consumption of total free sugars at or above 20 TE% to examine this threshold in our cohort.

In an American analysis, the lowest quintile of sugar intake was associated with the highest CVD mortality in men [31]. This study examined men and in women 50 to 71 years of age from the National Institute of Health-American Association of Retired Persons cohort, deriving sugar intakes from a food frequency questionnaire (grams per 1000 kcals total energy). Associations of added sugars and CVD mortality were null in women; in men, across quintiles 2 through 5, there was an approximately 10% lower hazards of CVD mortality than in the first quintile. We estimated that added sugars in this quintile averaged 2.5 TE%, a very low intake that may reflect an inadequate diet; this differs from our examination of differences at or above vs. below a 10 TE% threshold.

While ‘tolerated’ sugar intake may differ from geographic region to geographic region, it also appears to vary with age group and outcome considered. In an analysis that we performed in younger females from the same source population as the present analyses (CCHS 2004–2005 cohort), [32] we determined that those consuming free sugars from solid sources at or above 5 TE% had lower odds of overweight and gestational diabetes mellitus (GDM) than those consuming below this threshold. Some other studies have also reported better weight and diabetes outcomes with higher intakes of some sugar-sweetened foods. This includes an NHANES analysis in children evaluating associations with BMI [33] and an overview of meta-analyses assessing associations with type 2 diabetes [12]. Associations with free sugars may thus differ by outcome and/or population.

We did not detect conclusive differences specifically in relationship to higher intakes of free sugars from liquid sources. The American NHANES analyses [7] that identified associations between total added sugars and fatal CVD outcomes reported 29% higher hazards of fatal CVD with consumption of 7 or more sugar-sweetened beverages (360 ml) per week compared to 1 or less. In the previously-cited Swedish study, [30] those consuming more than 8 servings per week of sugar sweetened beverages had 19% increased hazards of stroke than those consuming less than 1 serving per week. We did not examine sugar-sweetened beverages in isolation but rather free sugars from liquid sources at or above vs below a 5 TE% threshold. Less than 13% of participants consumed soft drinks, with higher proportions consuming fruit juices, sauces, and salad dressings. A recent systematic review [34] indicated limited evidence for conclusive associations between 100% fruit juice consumption and fatal CVD while an analysis of the Dutch component of the European Prospective Investigation into Cancer and Nutrition (EPIC) cohort found 17% lower hazards of CVD with up to 7 glasses/week fruit juice compared to no consumption [35]. Notably in our analyses, daily fruit juice consumption was associated with lower hazards for CVD in younger men (Additional file 1).

A pervasive challenge in nutritional epidemiological studies is the ascertainment of intake [36, 37]. We based our analyses on a single 24-h dietary recall. There is certainly day-to-day variation in intakes. Indeed, among the one third of participants who completed a secondary 24-h dietary recall, classification of intakes of free sugars was moderately rather than highly correlated between recalls, with 30 to 40% switching categories. In a recent analysis restricted to those who underwent two dietary recalls, investigators did not capture any associations between sugars and CVD outcomes [38]. We suspect that this may have partly been due to the smaller sample included in their analyses, which may have limited the scope for performing sex or age group-specific analyses. Of note, the NHANES analysis that examined fatal CVD outcomes [7] used  food frequency questionnaires with a 7-day recall for the main analyses, but obtained similar results in a sensitivity analysis using a single 24-h dietary recall. This supports our decision to use a single 24-h dietary recall. To mitigate the potential impact of day-to-day variations in dietary intake, we did adjust for self-reported impressions of variations from usual intake. Similar to self-reported dietary intakes, adjustments for physical activity levels are limited by the imprecision of interview-based assessments of physical activity, subject to over reporting and recall difficulties. With respect to outcome measures, we acknowledge that the health administrative data used do not incorporate blood tests (e.g., cardiac enzymes), electrocardiograms, or imaging (echocardiograms, magnetic resonance imaging or computed tomography) that could corroborate CVD diagnoses; however, to reduce the scope for misclassification, we applied validated ICD code-based definitions for CVD. We did not have access to CCHS survey weights and thus caution should be exercised when our findings are extrapolated to the Canadian population as a whole; we emphasize, however, that our focus was on estimates of associations rather than population-level estimates of mean intakes. Lastly, among younger men and women (aged 35–55), the total number of CVD events (154 events and 72 events, respectively) and incidence rates were relatively low; we speculate that we had insufficient statistical power to demonstrate conclusive associations among these younger groups due to the lack of CVD events occurring before the age of 55. Strengths of our study include the use of linked databases that allow capture of both fatal and nonfatal CVD, and detailed baseline information on important co-variates, including food security and various dietary factors.

## Conclusions

Consideration of our findings in relationship with the wider literature suggests that associations of free sugars with adverse outcomes may differ in relationship to the outcome of interest, the age and sex of the study population, and even geographic location, which itself may impact food quality, physical activity, culture, and other factors. Our findings do indicate that from a CVD prevention standpoint in those 55 to 75 years of age, there may be benefits from consuming less than 5 TE% as free sugars from solid sources.

## Supplementary Information

**Additional file 1. **Adjusted associations with baseline characteristics other than free sugars with CVD.

## Data Availability

The data made available for linkage are only accessible through the Canadian Research Data Centre Networks with permission from Statistics Canada. The data that support the findings of this study are not available from the authors as they do not hold the data files. All analyses were conducted by the authors at a Statistics Canada Research Data Centre, a secure physical environment available to accredited researchers in Canada for research purposes. These centres are located on university campuses across Canada and are staffed by Statistics Canada employees. For further information, please see https://www.statcan.gc.ca/eng/microdata.

## Abbreviations

LOESS: Locally estimated scatterplot smoothing
SD: Standard deviation
%FS: Percent of total sugars component calculated to be free sugars
b.f.: Butter fat
kcals: Calories
TE%: % Total energy
CVD: Cardiovascular disease
HR: Hazard ratio
N: Number
WHO: World Health Organization
NHANES: National Health and Nutrition Examination Survey
CCHS: Canadian Community Health Survey
DAD: Discharge Abstract Database
CMDB: Canadian Mortality Database
ICD: International Classification of Disease
BNS: Bureau of Nutritional Sciences
TE: Total energy
BMI: Body mass index
EPIC: European Prospective Investigation into Cancer and Nutrition
