# Supplementary material for: Associations of free sugars from solid and liquid sources with cardiovascular disease: a retrospective cohort analysis
Source: BMC Public Health. 2023 Apr 24;23:756. doi: 10.1186/s12889-023-15600-3 (PMC10124057; doi:10.1186/s12889-023-15600-3)
Supplement: Supplementary file 1 — Additional file 1. Adjusted associations with baseline characteristics other than free sugars with CVD. [file 12889_2023_15600_MOESM1_ESM.docx]

|  | 55 to 75 years | | 35 to 55 years | |
| --- | --- | --- | --- | --- |
|  | **Men** | **Women** | **Men** | **Women** |
| Baseline characteristics^1^ | **Adjusted HR**  **(95% CI)** | **Adjusted**  **HR (95% CI)** | **Adjusted**  **HR (95% CI)** | **Adjusted**  **HR (95% CI)** |
| Obesity^2^ | 1.94  (1.38 – 2.72) | 1.02  (0.74 – 1.41) | 1.24  (0.78 – 1.97) | 1.34  (0.73 – 2.47) |
| Overweight^2^ | 1.37  (1.01 – 1.87) | 1.08  (0.80 – 1.45) | 1.20  (0.77 – 1.85) | 0.80  (0.42 – 1.55) |
| Age, years | 1.06  (1.04 – 1.08) | 1.09  (1.07 – 1.11) | 1.13  (1.09 – 1.17) | 1.07  (1.02 – 1.13) |
| Europid | 1.27  (0.74 – 2.20) | 0.99  (0.57 – 1.75) | 0.68  (0.38 – 1.22) | 0.85  (0.35 – 1.93) |
| Immigrant | 0.83  (0.60 – 1.15) | 0.77  (0.53 – 1.10) | 0.67  (0.34 – 1.31) | 0.52  (0.17 – 1.55) |
| Food insecure | 1.35  (0.72 – 2.54) | 1.00  (0.55 – 1.81) | 2.08  (1.24 – 3.48) | 1.52  (0.78 – 2.99) |
| Rural residence | 0.94  (0.73 – 1.21) | 0.90  (0.68 – 1.19) | 1.02  (0.70 – 1.47) | 0.75  (0.42 – 1.41) |
| Active | 0.89  (0.70 – 1.12) | 0.84  (0.65 – 1.09) | 0.85  (0.60 – 1.20) | 0.98  (0.58 – 1.63) |
| Post-secondary education | 0.81  (0.64 – 1.03) | 0.88  (0.68–1.13) | 1.14  (0.80 – 1.62) | 0.96  (0.56 – 1.63) |
| Smoking | 1.57  (1.19 – 2.08) | 2.25  (1.69 – 3.00) | 1.48  (1.04 – 2.12) | 1.51  (0.86 – 2.65) |
| Amount of food intake reported in last 24-hr compared to usual intake (reference: typical intake) | Much more:  0.62  (0.34 – 1.11)  Much less:  1.06  (0.76 – 1.49) | Much more:  1.21  (0.79 – 1.85)  Much less:  0.95  (0.66 – 1.38) | Much more:  0.78  (0.36 – 1.70)  Much less:  1.28  (0.85 – 1.93) | Much more:  1.19  (0.50 – 2.83)  Much less:  0.83  (0.43 – 1.60) |
| Total energy intake (per 100 kcal) | 1.01  (0.99 – 1.02) | 1.00  (0.99 – 1.00) | 1.00  (0.99 – 1.00) | 1.00  (0.99 – 1.00) |
| Non-sugar carbohydrates | 1.01  (0.99 – 1.03) | 1.00  (0.98 – 1.02) | 0.98  (0.96 – 1.01) | 1.00  (0.97 – 1.04) |
| Saturated fat, TE% | 0.98  (0.95 – 1.02) | 0.99  (0.95 – 1.02) | 0.94  (0.89 – 1.00) | 0.92  (0.84 – 0.99) |
| Monounsaturated fat intake, TE% | 1.05  (1.00 – 1.09) | 0.98  (0.94 – 1.03) | 0.99  (0.93 – 1.05) | 1.02  (0.94 – 1.12) |
| Polyunsaturated fat intake, TE% | 0.99  (0.92 – 1.06) | 1.02  (0.96 – 1.09) | 1.03  (0.95 – 1.12) | 1.00  (0.88 – 1.14) |
| Protein, %TE | 1.02  (0.99 – 1.04) | 1.02  (1.00 – 1.05) | 0.99  (0.97 – 1.02) | 0.96  (0.91 – 1.01) |
| Sodium intake, g | 1.00  (0.94 – 1.08) | 1.05  (0.95 – 1.15) | 1.06  (0.98 – 1.14) | 0.99  (0.82 – 1.18) |
| Potassium intake, g | 0.98  (0.89 – 1.08) | 0.89  (0.78 – 1.01) | 1.08  (0.95 – 1.22) | 1.23  (0.99 – 1.54) |
| Fibre, g | 1.00  (0.99 – 1.01) | 0.99  (0.98 – 1.01) | 0.98  (0.96 – 1.00) | 0.98  (0.95 – 1.02) |
| Daily fruit juice | 1.20  (0.94 – 1.53) | 0.87  (0.67 – 1.13) | 0.68  (0.46 – 1.00) | 1.29  (0.74 – 2.25) |
| Fruits/vegetables ≥5 servings/day | 0.89  (0.66 – 1.18) | 1.02  (0.77 – 1.35) | 0.75  (0.46 – 1.23) | 1.04  (0.57 – 1.92) |
| Hypertension | 1.31  (1.02 – 1.67) | 1.58  (1.23 – 2.03) | 1.48  (0.95 – 2.31) | 1.82  (0.95 – 3.52) |
| Intestinal/Stomach Ulcers | 1.30  (0.67 – 2.51) | 0.84  (0.45 – 1.55) | 3.17  (1.51 – 6.65) | 3.14  (1.39 – 7.08) |
| Cancer | 1.45  (0.87 – 2.42) | 1.55  (0.84 – 2.87) | 0.92  (0.13 – 6.66) | 1.83  (0.43 – 7.89) |
| Bowel Disorder | 1.00  (0.46 – 2.16) | 1.81  (1.15 – 2.83) | 1.14  (0.28 – 4.69) | 1.72  (0.75 – 3.94) |

TE%, total energy; g, grams; 24-hr, 24-hour

**Table Legend**: Overweight and obesity were conclusively associated with higher CVD hazards among older men (55-75 years of age). While daily fruit juice consumption demonstrated associations that were indicative (but not conclusive) of lower CVD hazards among younger men (35-55 years of age), food insecurity demonstrated conclusive associations with higher CVD hazards among this group. Age was conclusively associated with CVD across all demographic groups. Smoking also demonstrated conclusive associations with CVD across all groups, with the exception of younger women (findings are indicative but not conclusive).

^1^The multivariable model examined associations with baseline variables at the above vs. below 5 TE% threshold of free sugar intake from solid and liquid sources. The reference group for each dichotomous variable is represented by the opposing category (e.g. non-Europid, non-immigrant). Men and women with missing data for covariates other than overweight/obesity (i.e. Europid, immigrant, post–secondary education, etc.) were collectively unavailable for <1% of the study cohort and thus excluded from our regression models.

^2^BMI computation was based on directly measured anthropometric data in 61% of men 55 to 75 years, 67.5% of women 55 to 75 years, 59% of men 35 to 55 years, and 63% of women 35 to 55 years. BMI data was missing for 3.2% of men 55 to 75 years, 4.3% of women 55 to 75 years, 3% of men 35 to 55 years, and 5.2% of women 35 to 55 years; these individuals were excluded from the regression models. For the remainder, BMI was computed using self–reported data. Overall, there was under 5% missingness across all variables entered the regression model; those with missing data were excluded from our regression models.
